# Flurbiprofen used in one-lung ventilation improves intraoperative regional cerebral oxygen saturation and reduces the incidence of postoperative delirium

**DOI:** 10.3389/fpsyt.2022.889637

**Published:** 2022-08-31

**Authors:** Liang Shen, Jia-qi Chen, Xin-lu Yang, Ji-cheng Hu, Wei Gao, Xiao-qing Chai, Di Wang

**Affiliations:** ^1^Department of Anesthesiology, Anhui Provincial Hospital Affiliated to Anhui Medical University, Hefei, China; ^2^Pain Clinic, Department of Anesthesiology, First Affiliated Hospital of USTC, Division of Life Sciences and Medicine, University of Science and Technology of China (USTC), Hefei, China

**Keywords:** flurbiprofen, one-lung ventilation, regional cerebral oxygen saturation, postoperative delirium, thoracic surgery

## Abstract

**Background:**

We previously demonstrated that flurbiprofen increased arterial oxygen partial pressure and reduced intrapulmonary shunts. The present study aims to investigate whether flurbiprofen improves intraoperative regional cerebral oxygen saturation (rScO_2_) and reduces the incidence of postoperative delirium (POD) in elderly patients undergoing one-lung ventilation (OLV).

**Methods:**

One hundred and twenty patients undergoing thoracoscopic lobectomy were randomly assigned to the flurbiprofen-treated group (*n* = 60) and the control-treated group (*n* = 60). Flurbiprofen was intravenously administered 20 minutes before skin incision. The rScO_2_ and partial pressure of arterial oxygen (PaO_2_) were recorded during the surgery, and POD was measured by the Confusion Assessment Method (CAM) within 5 days after surgery. The study was registered in the Chinese Clinical Trial Registry with the number ChiCTR1800020032.

**Results:**

Compared with the control group, treatment with flurbiprofen significantly improved the mean value of intraoperative rScO_2_ as well as the PaO_2_ value (P < 0.05, both) and significantly reduced the baseline values of the rScO_2_ area under threshold (AUT) (*P* < 0.01) at 15, 30, and 60 min after OLV in the flurbiprofen-treated group. After surgery, the POD incidence in the flurbiprofen-treated group was significantly decreased compared with that in the control group (*P* < 0.05).

**Conclusion:**

Treatment with flurbiprofen may improve rScO_2_ and reduce the incidence of POD in elderly patients undergoing thoracoscopic one-lung ventilation surgery for lung cancer.

**Clinical trial registration:**

http://www.chictr.org/cn/, identifier ChiCTR1800020032.

## Introduction

One-lung ventilation (OLV) refers to the mechanical separation of the two lungs to allow ventilation of only one lung, while the other lung is compressed by the surgeon or allowed to passively deflate (1). This non-physiological ventilation approach is a standard approach to facilitate surgical exposure for pulmonary and other thoracic surgeries by using either a double-lumen tube or bronchial blocker (2). However, hypoxemia is one of the most common complications associated with OLV, occurring in 7–28% of individuals subjected to OLV (3, 4). It is thought that hypoxemia during OLV is related to ventilation/perfusion disturbance due to intrapulmonary shunts (5, 6). Although hypoxic pulmonary vasoconstriction (HPV) allows redirection of blood flow into the ventilated lung, approximately 4–10% of patients still experience an oxygen saturation of <90% (1, 7, 8).

Brain cellular function may be impaired or damaged by the reduction in oxygen delivery under this level of peripheral oxygen desaturation (9). Brain oximetry based on cerebral near-infrared spectroscopy (NIRS) enables continuous and noninvasive measurement of cerebral tissue oxygen saturation (SctO_2_) or regional cerebral oxygen saturation (rScO_2_) (10, 11). The incidence of cerebral oxygen desaturation, depicted as a decrease in SctO_2_ of more than 15% from the baseline level, has been reported to be as high as 70–100% in thoracic surgical patients undergoing OLV (7, 12). Cerebral hypoxia is a well-established risk factor for postoperative delirium (POD), which is an acute fluctuating brain dysfunction characterized by inattention, disorganized thinking, and altered levels of consciousness (13, 14). The reported incidence of POD ranges from 7 to 23% in patients who receive OLV (15, 16). It has been reported that cerebral desaturation, defined by 90% baseline for left SctO_2_, may be associated with an increased risk of POD in thoracotomy with OLV (17).

We have demonstrated that treatment with flurbiprofen reduced the Qs/Qt ratio and further increased the PaO_2_ level during OLV, possibly due to the upregulation of the vasoactive agent thromboxane B2 (TXB2)/6-keto-prostaglandin F1α (6-K-PGF1α) ratio (18). However, whether flurbiprofen can further alleviate cerebral desaturation and reduce POD incidence is largely unclear. In the present study, we aimed to determine whether flurbiprofen alleviates the reduction in the intraoperative rScO_2_ value and decreases the incidence of POD.

## Patients and methods

### Trial design

This is a prospective, randomized, double-blind and controlled trial implemented in First Affiliated Hospital, University of Science and Technology of China (USTC). This study was conducted in accordance with the Declaration of Helsinki and approved by the Institutional Ethics Committee. All the patients were informed of the full details of the study protocol and signed the informed consent from. The study was registered in the Chinese Clinical Trial Registry with the number ChiCTR1800020032.

### Randomization and blinding

The patients were randomized to either the flurbiprofen treatment group (Group F) or the control group (Group C) before entering the operation room. An allocation sequence was created by a computer-generated list. Allocation concealment was implemented by using sequentially numbered, opaque, sealed envelopes. Randomization and drug preparation were performed by an independent investigator who was not involved in the administration of anesthesia. Flurbiprofen and fat emulsions of the same appearance were sent to the anesthesiologist in an unmarked syringe before intravenous injection. Data collection was performed by another independent researcher involved in the administration of anesthesia. Data were collected in a blinded manner by the study patients, anesthesiologists, other researchers, and statisticians.

### Patients

**Figure 1:**
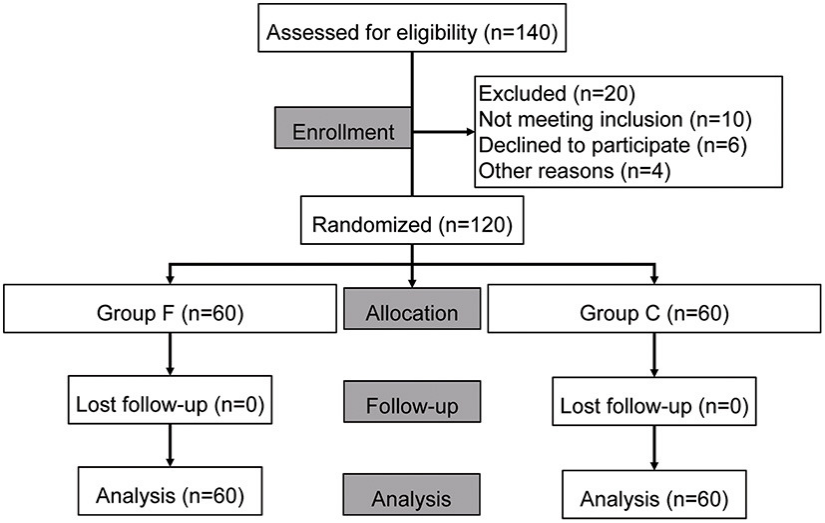
Flow chart of study.

From June 2020 to April 2021, patients who were scheduled for elective pulmonary lobectomy and underwent video-assisted thoracoscopic surgery (VATS) were assessed before the study. Figure 1 shows the flow diagram of participant recruitment. The inclusion criteria were as follows: (1) age between 65 and 75 years; (2) American Society of Anesthesiologists (ASA) status II-III; and (3) anticipated duration of one-lung ventilation >60 min.

Exclusion criteria were as follows: (1) severe impairment of respiratory function (forced expiratory volume in 1 s of <50% of the predicted values); (2) presence of contraindications for using flurbiprofen or intralipid; (3) treatment with NSAIDs drugs within 1 month before surgery; (4) scored<24 on the Mini-mental State Examination (MMSE) or who were unable to complete baseline cognitive assessment; (5) history of smoking, alcohol or drug abuse; (6) difficulty to maintain oxygenation with one-lung ventilation intraoperatively; (7) duration of one-lung ventilation was smaller than 60 min; (8) unable to collect data due to the cerebral oximeter machine malfunction; (9) researchers believe that other situations do not meet the conditions of this study. For example, VATS was intraoperatively converted to an open thoracotomy procedure.

### Treatment

The participants were randomized to either the flurbiprofen treatment group (Group F) or the control group (Group C). Flurbiprofen 100 mg (50 mg/5 ml, Beijing Tide Pharmaceutical, China) was in Group F, and the placebo (Intralipid, Chengdu Huarui Pharmaceutical, China) was in Group C. The trial drug was dissolved in 100 ml of normal saline and intravenous drip within 15 min. All subjects received the drug (flurbiprofen or placebo) 20 min before incision. The length of the trial drug to OLV start was approximately 30 min.

### General anesthesia

No premedication was used prior to surgery. General anesthesia was initiated with intravenous 0.05 mg/kg midazolam, 2 mg/kg propofol, 0.4 μg/kg sufentanil, and 1.0 mg/kg rocuronium. After the induction of anesthesia, an endobronchial tube was inserted and confirmed by bronchoscopy. Intubation was performed using a double-lumen tube. An additional 0.15 μg/kg sufentanil was given before the incision. Subsequently, intraoperative anesthesia was maintained with a continuous infusion of propofol (4–8 mg/kg/h) and remifentanil (0.05–0.2 μg/kg/min) to achieve a target bispectral index (BIS) value between 40 and 50. Cisatracurium was administered intermittently as required during the surgery. All patients were mechanically ventilated to maintain 35–45 mmHg ETCO_2_. Patients were mechanically ventilated in constant-flow volume-controlled mode following the protocol with a tidal volume of 6 to 8 mL/kg (two-lung ventilation) or 4–6 mL/kg (one-lung ventilation), inspiratory-to-expiratory time ratio of 1:2, and positive end-expiratory pressure 5 cm H_2_O. Pulse oxygen saturation was maintained above 92%. All surgical operations were performed by the same surgeon team, without any additional administration of local anesthetic by surgeons. Postoperative analgesia was regularly conducted using patient-controlled analgesia (PCA): Sufentanil was delivered at a rate of 2 μg/hr, with a 1.5 μg bolus and lockout interval of 15 min for breakthrough pain. Tramadol (50 mg) was provided for rescue analgesia if the visual analog scale (VAS) score was ≥4.

### Monitoring indicators

Throughout the perioperative period, electrocardiography (ECG), heart rate (HR), SpO_2_, blood pressure, and CVP were continuously monitored. A bispectral index (BIS) sensor (Aspect Medical Systems, Inc., USA) applied to the forehead was used to monitor the depth of anesthesia, and the BIS value was kept at 40–50. The nasopharyngeal temperature was maintained at 36.3–37.2°C. Phenylephrine was given at a bolus of 25 μg if mean blood pressure (MBP) decreased to <80% of the preoperative baseline or systolic blood pressure decreased to <90 mmHg; atropine (0.3 mg) was given if HR decreased to <50 beats/min. If MBP or HR increased by >20% of the preoperative baseline, patients received fentanyl (0.05 mg) followed by perdipine (0.2 mg) or esmolol (10 mg). These treatments were repeated if necessary.

### rScO_2_ monitoring

All patients were monitored with the INVOS 5100C cerebral oximeter before anesthesia induction until extubation. A fiberoptic sensor was positioned on each side on the forehead of the patients and covered by an opaque plastic patch to prevent ambient light. Baseline absolute rScO_2_ values were taken in the awake patient after 2 min of breathing 100% oxygen through a face mask. The screen of the cerebral oximeter was covered with an opaque bag to blind the rScO_2_ data to anesthesia providers and surgeons for monitoring. The rScO_2_ data were recorded at 30-s intervals on the device's accessory disk drive for later analysis. With the whole rScO_2_ data of each subject, the baseline, mean and minimum absolute rScO_2_ values of both sides during surgery were recorded, calculated and sifted. According to reference, cerebral desaturation was defined as either of the following two situations: (1) a baseline absolute value over 50%, rScO_2_ reduced to <75 percent of baseline; (2) or a baseline absolute value <50%, rScO_2_ reduced to <80 percent of baseline (19). Thus, in this trial, the rScO_2_ threshold was defined as 75% of the baseline absolute value if the baseline value was ≥50%, and if the baseline value was <50%, the rScO_2_ threshold was defined as 75% of the baseline value. The area under the threshold (AUT-rScO_2_) was also calculated. AUT was based on the following formula: AUT (present) = AUT (past) + (rScO_2_ threshold- rScO_2_ value) × sample rate. The AUT was 0 if the rScO2 value was above the defined rScO_2_ threshold (20).

### Neuropsychological tests

The Confusion Assessment Method (CAM) is a widely used and well-validated screening tool for delirium with a sensitivity of 94% (95% confidence interval [CI] = 91–97%) and specificity of 89% (95% CI = 85–94%) and has been successfully adapted for use in the intensive care unit (ICU) setting (the CAM-ICU was used where appropriate) (21, 22). POD was defined as any episode of delirious symptoms within five postoperative days. Delirium was assessed twice daily, between 06:00–08:00 and 18:00–20:00, using the Chinese version of the Confusion Assessment Method (CAM) in non-intubated patients and the CAM-ICU in intubated patients. (17). The research personnel who were responsible for delirium assessment participated in a 4-h training session with the following agenda: (1) an introduction to the symptoms, diagnosis, and treatment of delirium, (2) a lecture on how to use CAM and CAM-ICU for delirium assessments, and (3) a simulation training course with a quiz at the end of training. All trainees were required to answer all quiz questions correctly. Research personnel who were responsible for out-come assessment were not allowed to access patient data collected during surgery (17).

### Sample collection

Blood samples were collected from arterial catheters before anesthesia, 15 min after double-lung ventilation (supine position), 15, 30, and 60 min after OLV, and 30 min after re-expansion of the collapsed lung. The samples were tested by blood gas analyses. The Qs/Qt ratio was determined from the following formula: (CCO_2_ – arterial oxygen content)/ (CCO_2_ – mixed venous oxygen content), where CCO_2_ = end-pulmonary capillary oxygen content.

### Other outcomes

Baseline blood pressure, baseline heart rate, operation time, anesthesia time, OLV time, intraoperative blood loss, urine volume, infusion volume, length of hospital stay, length of stay in the ICU, pulmonary infection rate, chest tube removal time, visual analog score, and death rate 1 month after surgery were recorded for every patient.

### Sample capacity

Few studies have tested the effect of flurbiprofen on cerebral oxygen saturation in one-lung ventilation. However, based on our pre-experiment outcome, we considered a difference of 3% for the mean rScO_2_ between both groups and a pooled SD of 7% of the means. With a power (β) of 0.95 and a 5% significance level, a minimum sample size of 59 patients for each group was estimated. Considering a drop-out rate of 20%, 70 subjects whose elective pulmonary lobectomy was undergoing VATS with an ASA status of II-III were registered in each group (140 patients in total).

### Statistical analysis

Data were analyzed using SPSS 16.0 software. Values are expressed as the mean ± standard deviation (SD) or n (%). The comparison of the two groups of normal data was performed using one-way analysis, the comparison of two groups of nonnormal data was performed using the independent sample *t*-test and the Mann–Whitney *U* test, and the comparison of count data was performed by the chi-square test. Multiple reading data were analyzed by repeated-measures analysis of variance. A *P* < 0.05 was considered statistically significant, and a *P* < 0.01 was considered highly statistically significant.

## Results

### Patient demographics

One hundred and twenty patients aged between 65 and 75 years underwent elective pulmonary lobectomy for this study. There were 60 patients in each group. The characteristics of the 120 patients are summarized in Table 1. There were no significant differences in age, sex, ASA grade, year of education, MMSE score, hemoglobin before surgery, clinical characteristics, or surgery side between the two groups (*P* >0.05).

**Table 1:** Patient characteristics.

**Characteristic**	**Group C (*n* = 60)**	**Group F (*n* = 60)**	***p* Value^a^**
Age (years)	68.38 ± 3.08	68.63 ± 2.91	0.9310
**Gender**		0.6011	
Male	38 (63.3%)	40 (66.7%)	
Female	22 (36.7%)	20 (33.3%)	
ASA physical status			0.7158
**II**	11 (18.3%)	13 (21.7%)	
**III**	49 (81.7%)	47 (78.3%)	
BMI (kg/m^2^)	24.78 ± 2.48	25.28 ± 2.29	0.4299
**Education (years)**			0.4324
≤9 years	52 (86.7)	52 (83.3)	
>9 years	8 (13.3)	10 (16.7)	
MMSE before surgery	27.52 ± 2.018	27.19 ± 1.966	0.8032
**Clinical characteristics**			
History of stroke	8 (13.3%)	10 (16.7%)	0.6291
Hypertension	26 (43.3%)	24 (40.0%)	0.6932
Diabetes	15 (25.0%)	18 (30.0%)	0.5294
**Surgery side**			0.5104
Left	25 (41.7%)	28 (46.7%)	
Right	35 (58.3%)	32 (53.3%)	
Hemoglobin (g/dl)	12.64 ± 1.42	12.62 ± 1.74	0.9742

Values are n (%) or mean ± SD.

a The comparison among the two groups of normal data using one-way analysis, the comparison of count data by chi-square test.

ASA, American Society of Anesthesiologists; BMI, body mass index; MMSE, Minimum Mental State Examination.

### Cerebral oxygen saturation values

**Figure 2:**
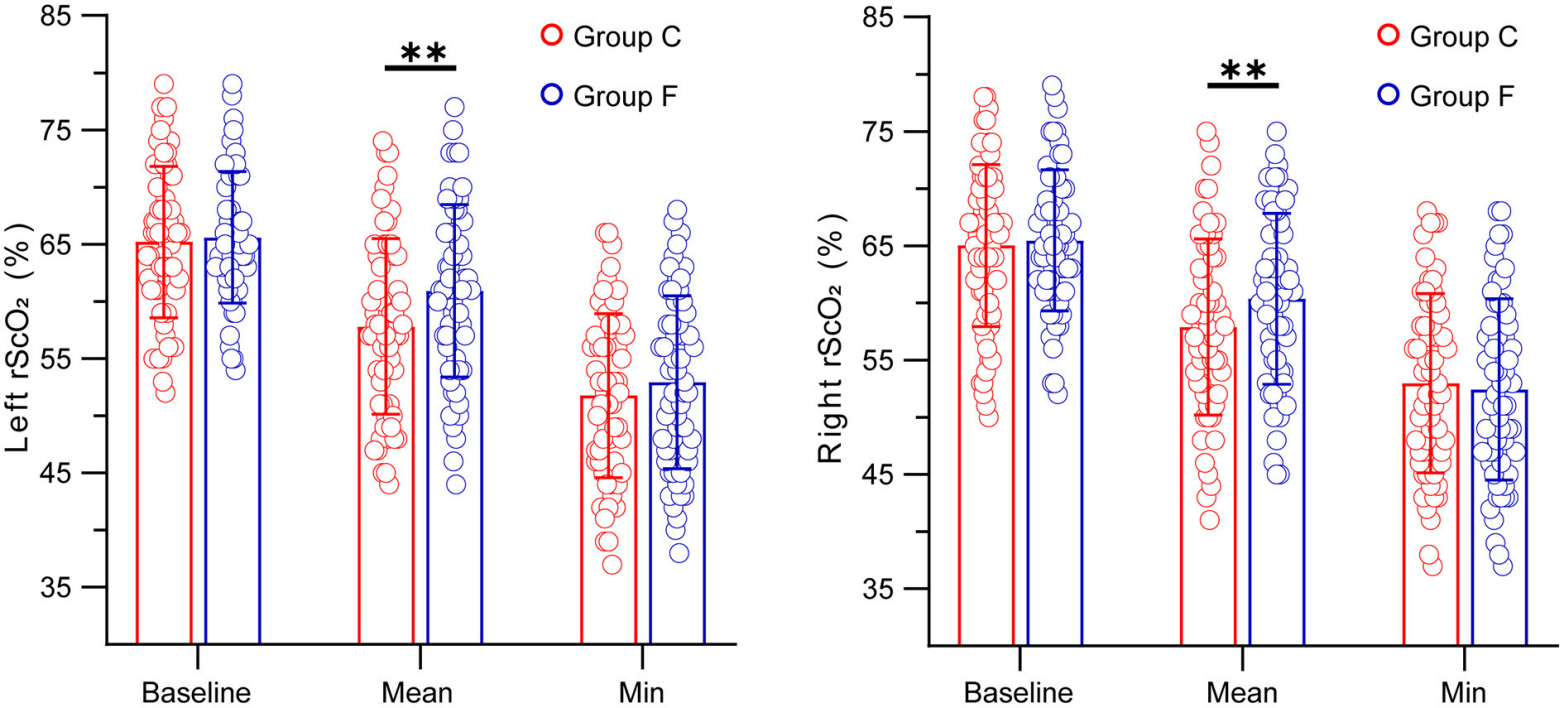
Baseline, mean and minimum of the left and right rScO_2_ values in Group F and Group C. All data are presented as mean ± SD, ^**^P < 0.01 vs Group C.

**Figure 3:**
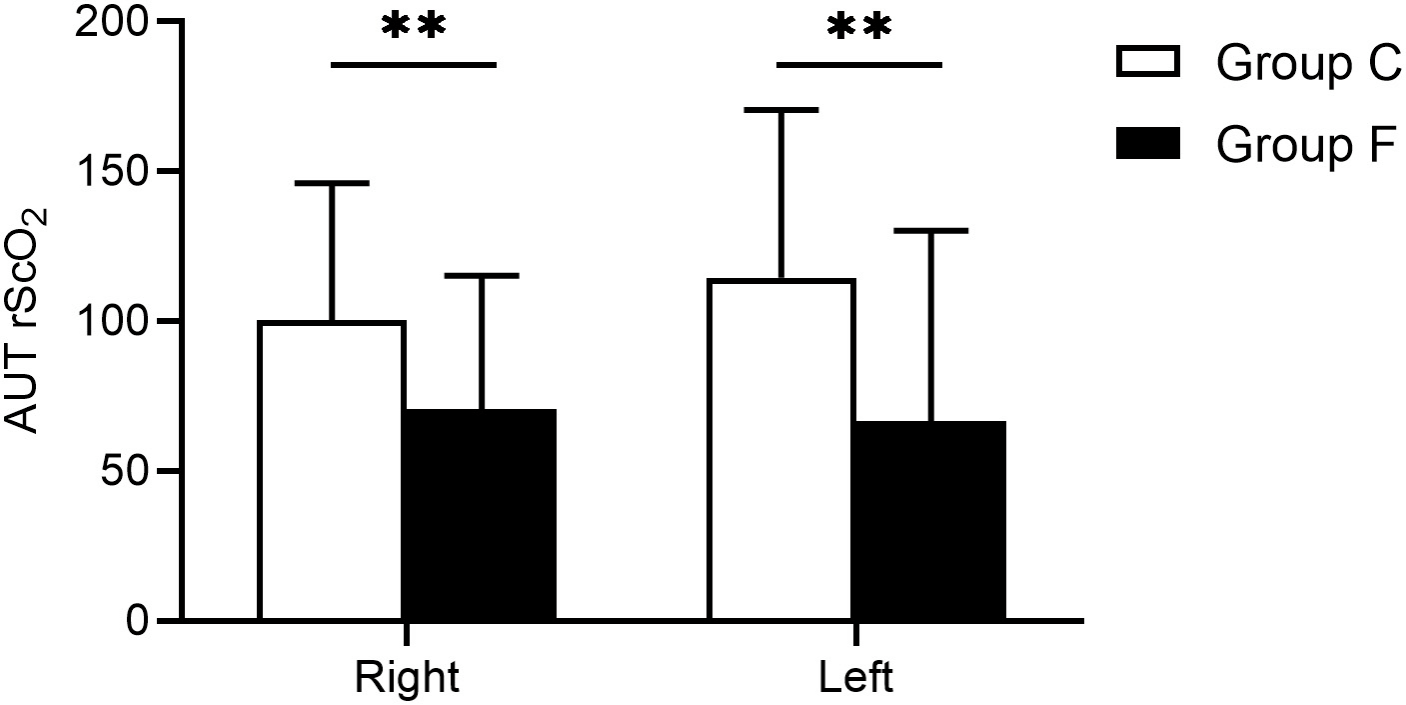
The left and right AUT rScO_2_ of baseline rScO_2_ values in Group F and Group C. All data are presented as mean ± SD, ^**^P < 0.01 vs. Group C. AUT rScO_2_, the area under the threshold of rScO_2_ readings.

**Figure 4:**
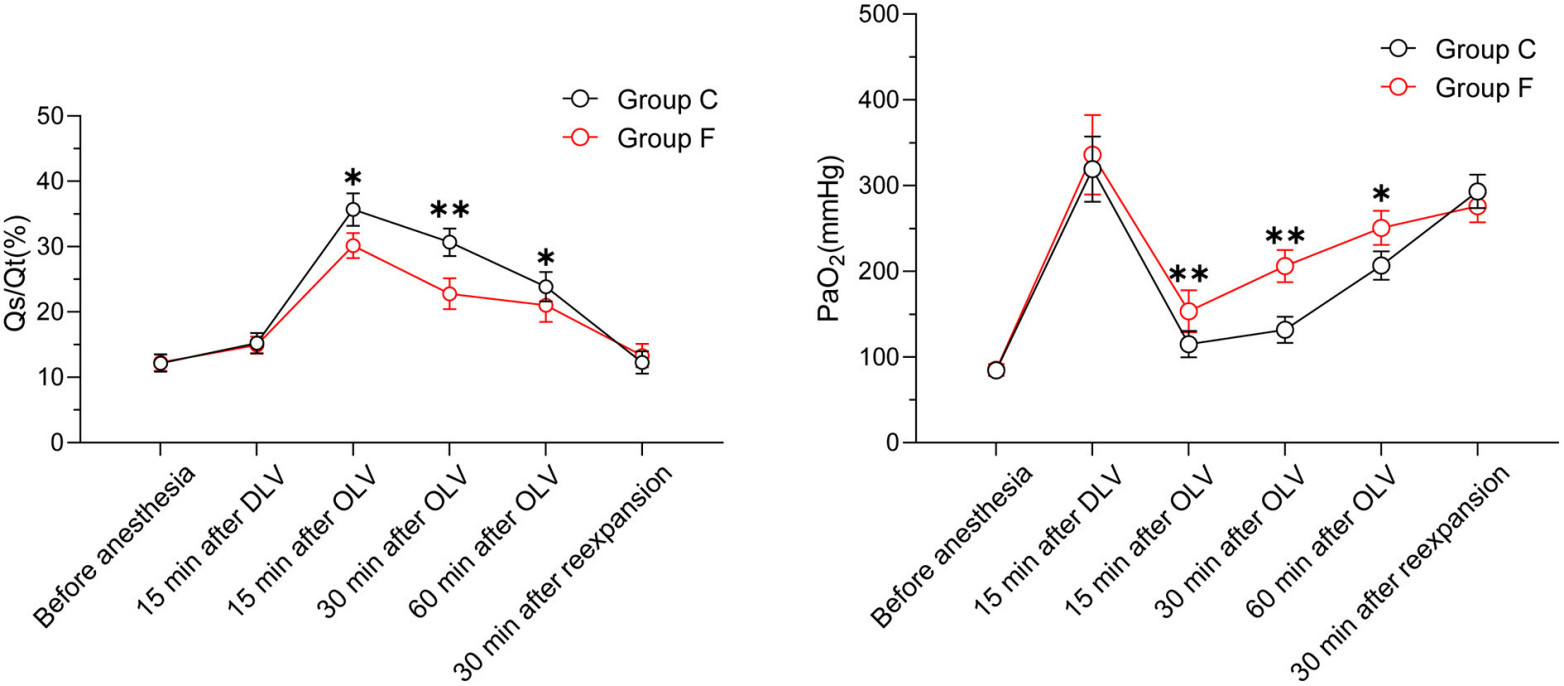
PaO_2_ and Qs/Qt values in Group F and Group C. All data are presented as mean ± SD, ^*^P < 0.05 and ^**^P < 0.01 vs. Group C.

In terms of the baseline values of rScO_2_ measured before anesthesia induction and the minimum values of rScO_2_ during a surgical operation, our findings showed that there were no significant differences between the flurbiprofen-treated group and the control group in either the right or left hemisphere (*P* > 0.05) (Figure 2). The mean value of rScO_2_ on both sides in the flurbiprofen-treated group was significantly higher than that in the control group (right hemisphere, *P* < 0.05; left hemisphere, *P* < 0.01) (Figure 2). In the flurbiprofen-treated group, the area under the threshold (AUT) of baseline rScO_2_, either in the right hemisphere or left hemisphere, was less than that in the control group, with significant differences (*P* < 0.01 both) (Figure 3). PaO_2_ levels throughout the intraoperative period in the flurbiprofen-treated group were higher than those in the control group (*P* < 0.05) (Figure 4).

**P < 0.01 vs Group C.

### POD incidence and other outcomes

**Figure 5:**
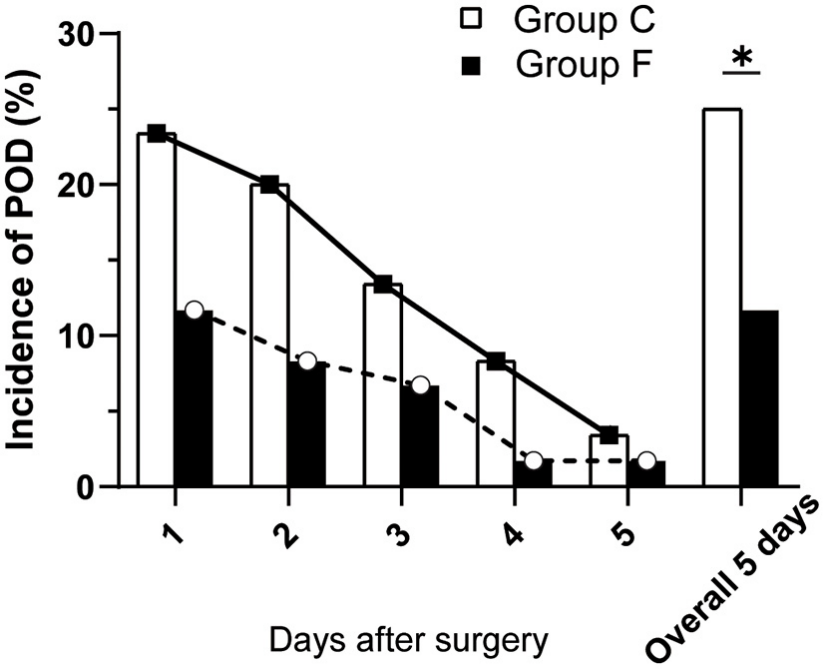
Incidence of POD in the two groups on postoperative days 1, 2, 3, 4, 5 and overall. All data are presented as n (%), ^*^P < 0.05 vs. Group C. POD, postoperative delirium.

Throughout the follow-up period after surgery, there were significant differences in POD development between the flurbiprofen-treated group and the control group at postoperative day 1 (7 vs. 14, *P* < 0.05), postoperative day 2 (5 vs. 12, *P* < 0.05), postoperative day 3 (4 vs. 8, *P* < 0.05), postoperative day 4 (1 vs. 5, *P* < 0.05), and postoperative day 5 (1 vs. 2, *P* < 0.05). The overall incidence of POD in the flurbiprofen-treated group was significantly lower than that in the control group (7 vs. 15, *P* < 0.05) (Figure 5). There were no significant differences in the baseline mean blood pressure, baseline heart rate, duration of anesthesia, duration of surgery, duration of OLV, phenylephrine and blood loss, urine output, fluid infusion during the surgery, chest tube removal time, lung infection rate, visual analog scale score, or ICU stay. However, the hospital stay of Group F was significantly shorter than that of Group C. No patient died at the one-month postoperative follow-up (Table 2). There were no significant differences in the intraoperative arterial blood oxygen saturation (SaO_2_) and arterial blood partial pressure of carbon dioxide (pCO_2_), as shown in Table 3.

**Table 2:** Perioperative data and postoperative outcomes.

**Information**	**Group C (*n* = 60)**	**Group F (*n* = 60)**	**p Value^a^**
Baseline MBP (mmHg)	92.25 ± 6.428	93.18 ± 5.792	0.4425
Baseline HR (beats/min)	69.37 ± 4.215	67.12 ± 6.14	0.5914
Duration of anesthesia (min)	247.73 ± 48.12	250.14 ± 45.73	0.9048
Duration of surgery (min)	186.17 ± 40.23	187.20 ± 46.56	0.9209
Duration of OLV (min)	129.33 ± 17.62	135.60 ± 21.20	0.5055
Phenylephrine (μg)	267.47 ± 129.77	283.23 ± 79.45	0.6831
Blood loss (ml)	287.83 ± 84.45	284.50 ± 58.34	0.8448
Urine output (ml)	480.33 ± 139.12	467.67 ± 114.64	0.7503
Fluid infusion (ml)	1,953.33 ± 257.95	2,015.17 ± 277.71	0.6897
Hospital-stay (days)	7.38 ± 2.27	6.52 ± 2.43	0.043
Chest tube removal (days)	4.11 ± 1.17	3.98 ± 1.30	0.574
visual analog scale	2.76 ± 0.87	2.45 ± 1.01	0.597
Lung infection^b^	10(16.66)	7(11.66)	0.198
1st month postoperative mortality rate	0	0	-
ICU-stay (days)	0.88 ± 2.37	0.87 ± 2.42	0.820

Values are n (%) or mean±SD.

a The comparison among the two groups of normal data using one-way analysis, the comparison of count data by chi-square test.

b Lung infection was defined through imaging and clinical manifestations 1 week after surgery.

MBP, mean blood pressure; HR, heart rate; OLV, one-lung ventilation.

**Table 3:** Intraoperative data.

**Information**	**Group C (*n =* 60)**	**Group F (*n =* 60)**	***p* Value^a^**
**Oxygen saturation (%)**			
Before anesthesia	98.3 ± 1.4	98.2 ± 0.8	0.9957
15 min after DLV	99.2 ± 0.5	99.3 ± 0.7	0.9957
15 min after OLV	97.1 ± 1.8	96.9 ± 1.5	0.8714
30 min after OLV	97.4 ± 1.1	97.3 ± 0.9	0.9957
60 min after OLV	97.8 ± 0.9	97.6 ± 0.6	0.8714
30 min after re-expansion	99.3 ± 0.4	98.9 ± 0.8	0.1894
**Partial pressure of carbon dioxide (mmHg)**			
Before anesthesia	40.1 ± 3.5	41.4 ± 2.4	0.1888
15 min after DLV	37.7 ± 4.7	37.8 ± 4.1	0.9899
15 min after OLV	43.5 ± 3.1	44.0 ± 3.7	0.9599
30 min after OLV	44.4 ± 2.3	44.1 ± 2.8	0.9972
60 min after OLV	45.3 ± 3.5	45.6 ± 2.6	0.9972
30 min after re-expansion	41.1 ± 2.1	42.0 ± 4.3	0.6023

Values are the mean±SD.

a The comparison among the two groups of normal data using One-way analysis, the comparison of count data by chi-square test.

DLV, double-lung ventilation; OLV, one-lung ventilation.

## Discussion

Hypoxemia remains challenging for intraoperative management, despite intervention including an increase in the inspired fraction of oxygen followed by a recruitment maneuvered and escalation of positive end-expiratory pressure (PEEP) to the ventilated lung (3, 6). Our study previously reported that intraoperative use of flurbiprofen increased arterial oxygen partial pressure and reduced intrapulmonary shunt (18). In the present study, our findings indicate that flurbiprofen may improve intraoperative rScO_2_ and further reduce the POD incidence in patients undergoing OLV. To the best of our knowledge, this is the first report to demonstrate the effects of flurbiprofen on intraoperative rScO_2_ and POD in elderly patients undergoing OLV.

Recently, an increasing number of published works have suggested NSAIDs (such as parecoxib, and flurbiprofen) that are commonly used for postoperative analgesia and can reduce POD after major noncardiac surgery in elderly patients (23, 24). However, the pharmacological mechanism underlying this beneficial action of NSAIDs on neuropsychiatric function is not entirely understood. It may involve the NSAID-elicited reduction of proinflammatory cytokines (TNF-α, IL-1β, and IL-6), which is thought to be primarily triggered by surgical operation and damage synapses and neurons and ultimately lead to POD *via* vagal afferents and by crossing the blood-brain barrier (25, 26). In addition to neuroinflammation, there are many other risk factors contributing to POD, such as age, sex, hypothermia, category of surgery, surgical position, duration of surgery, and mechanical ventilation. Given the complex etiology of POD, it is largely unknown whether flurbiprofen can in particular impact cerebral homeostasis, which has been shown to be closely related to the high risk of developing POD.

A variety of studies have indicated that the incidence of cerebral desaturation, defined as a decrease in SctO_2_ of more than 15% from the baseline level, can be as high as 70–100% in thoracic surgical patients (7). The cerebral desaturation during thoracic surgery might be due to a reduction in cerebral oxygen delivery that could be caused by an impairment either in arterial oxygen content or in cerebral blood flow that might also be related to a decrease in cardiac output. OLV together with the lateral decubitus position which is required for most cases of thoracic surgery, is accompanied by substantial physiological disturbances, including increases in pulmonary vascular resistance and pulmonary arteriovenous shunt, hypoxic pulmonary vasoconstriction, and reduction in alveolar-arterial oxygen tension. With the increase in pulmonary resistance during the collapse of the operated lung, the right ventricular cardiac output would be expected to decrease, especially under anesthesia where compensatory reflex mechanisms may be blunted (17). Furthermore, with impaired right ventricular performance, an increase in right-sided filling pressures could increase cerebral venous blood volume and affect cerebral saturation in this manner. Thus, these mechanisms could contribute to the decreases seen in cerebral oxygen saturation. It has been well-documented that flurbiprofen alleviates inflammation and pain by inhibiting cyclooxygenase (COX) activity and the synthesis of thromboxane A2 (TXA2)/prostaglandin I2 (PGI2), which are vasoactive agents that affect pulmonary arterial pressure, the Qs/Qt ratio, and PaO_2_. In the present study, flurbiprofen improved intraoperative rScO_2_ by increasing PaO_2_, probably owing to flurbiprofen-elicited adequate oxygen delivery.

Many studies have shown that the alteration of cerebral oxygen saturation is also of great clinical significance. For example, a study implied that once the maximum percentage decrease in rScO_2_ was more than 11%, the sensitivity and specificity of postoperative cognitive dysfunction occurrence were 86.5 and 77.8%, respectively. This suggests that if the maximum percentage decrease in rScO_2_ exceeds 11%, appropriate measures should be taken to increase rScO_2_ to prevent the risk of developing cerebral ischemia (27). Our study found that the maximum percentage decrease in rScO_2_ in single-lung ventilation was more than 11%. Furthermore, among patients undergoing coronary artery bypass grafting, maintenance of intraoperative monitored rScO_2_ values above a “safety threshold” was associated with a lower incidence of major organ dysfunction and a shorter hospital stay (28). Another study by Plachky et al. showed a positive relationship between decreased rScO_2_ during the anhepatic phase and a hypoxia/ischemia-induced increase in neuron-specific enolase, which is used as an index of cerebral damage, during orthotopic liver transplantation (29). Collectively, even a small improvement in cerebral oxygen saturation is clinically significant, and near-infrared spectroscopy determination has strong potential with clinical utility in differing settings. Therefore, we also cautiously compared the differences in hemoglobin, phenylephrine use, and intraoperative partial pressure of carbon dioxide, which may affect cerebral oxygen saturation, in the two groups, and the results were not significantly different. We consider that flurbiprofen may have improved the decrease in cerebral oxygen saturation during one-lung ventilation and this may contribute to the improvement of postoperative cognitive dysfunction.

Hypoxemia during one-lung ventilation triggers a concern that organ cellular function may be impaired or injured by the reduction in oxygen delivery (9, 12). Initially, perioperative cerebral oximetry monitoring primarily focused on cardiac surgery patients as this cohort had a known significant incidence of postoperative neurocognitive disorder and cerebral vascular incidents (22). As there was a growing recognition of the potential for intraoperative monitoring with cerebral oximetry, it was applied to non-cardiac surgery scenarios such as thoracic surgery, which often has an increased risk of intraoperative hypoxemia, and was one potential application of great interest. Although the validity needs to be further tested, multiple studies have shown a relationship between cerebral oxygen desaturations and neurocognitive deficits in patients undergoing thoracic surgical procedures (17, 30, 31). For example, Monique Roberts and colleagues indicated that intraoperative cerebral oxygen desaturations, frequent during one-lung ventilation, are significantly associated with worse early cognitive recovery, and a high risk of POD (31). Furthermore, it is important to understand how the severity of cerebral desaturation and POD is related and if there is a SctO_2_ threshold below which the risk of delirium is increased. In another study performed by Fan Cui and colleagues, cerebral desaturation defined by <90% baseline for left SctO_2_ and <85% baseline for right SctO_2_, may be associated with an increased risk of post-thoracotomy delirium (17). Our findings may support an additional mechanism by which flurbiprofen-elicited cerebral oxygen saturation might, at least in part, contribute to a reduction in POD.

There are several limitations. First, oxygen delivery is not solely dependent on saturation but rather must be considered in concert with hemoglobin level and, more importantly, cardiac output (8). Thus, whether hypoxemia reflected through peripheral oxygen saturation results in hypoxia is patient-dependent. Additionally, due to a lack of direct evidence, we do not know whether the participation of neuroinflammation underlies the beneficial actions of flurbiprofen (25, 26). Finally, this was a single-center study. The same team of performing surgeons and anesthetists ensured the standardization and consistency of the work. To add more evidence, multicenter studies are warranted to test the results and conclusions of our trial.

## Conclusions

Collectively, premedication with flurbiprofen may improve intraoperative rScO_2_ and reduce the incidence of POD in elderly patients undergoing thoracoscopic surgery with one-lung ventilation.

## Data availability statement

The original contributions presented in the study are included in the article/supplementary material, further inquiries can be directed to the corresponding author.

## Ethics statement

The Ethics Committee at the First Affiliated Hospital of USTC approved this prospective trial. Written informed consent was obtained from all patients recruited to the study, in accordance with the code of the Declaration of Helsinki.

## Author contributions

LS and J-qC: data curation and writing-original draft preparation. X-lY: writing-review and editing. J-cH and WG: analyze or synthesize study data. X-qC and DW: development or design of methodology. All authors contributed to the article and approved the submitted version.

## Funding

This work was supported by a grant from Anhui Provincial Key Research and Development Project Foundation (No. 1804h08020286).

## Conflict of interest

The authors declare that the research was conducted in the absence of any commercial or financial relationships that could be construed as a potential conflict of interest.

## Publisher's note

All claims expressed in this article are solely those of the authors and do not necessarily represent those of their affiliated organizations, or those of the publisher, the editors and the reviewers. Any product that may be evaluated in this article, or claim that may be made by its manufacturer, is not guaranteed or endorsed by the publisher.

## Abbreviations

rScO_2_: regional cerebral oxygen saturation
POD: postoperative delirium
OLV: one-lung ventilation
PaO_2_: partial pressure of arterial oxygen
CAM: Confusion Assessment Method
AUT: the area under threshold
HPV: hypoxic pulmonary vasoconstriction
NIRS: near-infrared spectroscopy
SctO_2_: cerebral tissue oxygen saturation
VATS: video-assisted thoracoscopic surgery
ASA: American Society of Anesthesiologists
MMSE: Mini-mental State Examination
BIS: bispectral index
PCA: patient-controlled analgesia
HR: heart rate
MBP: mean blood pressure
PEEP: positive end-expiratory pressure.
