# Corrigendum: Flurbiprofen used in one-lung ventilation improves intraoperative regional cerebral oxygen saturation and reduces the incidence of postoperative delirium

**DOI:** 10.3389/fpsyt.2023.1228369

**Published:** 2023-06-06

**Authors:** Liang Shen, Jia-qi Chen, Xin-lu Yang, Ji-cheng Hu, Wei Gao, Xiao-qing Chai, Di Wang

**Affiliations:** ^1^Department of Anesthesiology, Anhui Provincial Hospital Affiliated to Anhui Medical University, Hefei, China; ^2^Pain Clinic, Department of Anesthesiology, First Affiliated Hospital of USTC, Division of Life Sciences and Medicine, University of Science and Technology of China (USTC), Hefei, China

**Keywords:** flurbiprofen, one-lung ventilation, regional cerebral oxygen saturation, postoperative delirium, thoracic surgery

In the published article, there was an error regarding the affiliation for Di Wang. As well as having affiliation [2], they should also have [1].

The authors apologize for this error and state that this does not change the scientific conclusions of the article in any way. The original article has been updated.

